# A Comprehensive Review on Current Insights Into Epileptic Encephalopathy: Pathogenesis and Therapeutic Strategies

**DOI:** 10.7759/cureus.64901

**Published:** 2024-07-19

**Authors:** Rajvardhan Patil, Sunil Kumar, Sourya Acharya, Vineet Karwa, Suhail M Shaikh, Manjeet Kothari

**Affiliations:** 1 Internal Medicine, Jawaharlal Nehru Medical College, Datta Meghe Institute of Higher Education and Research, Wardha, IND; 2 Medicine, Jawaharlal Nehru Medical College, Datta Meghe Institute of Higher Education and Research, Wardha, IND

**Keywords:** personalized medicine, seizure management, therapeutic strategies, genetic factors, pathogenesis, epileptic encephalopathy

## Abstract

Epileptic encephalopathy (EE) represents a challenging group of disorders characterized by severe epilepsy and significant cognitive, behavioral, and neurological impairments. This comprehensive review aims to elucidate the current insights into the pathogenesis and therapeutic strategies for these disorders. Pathogenesis involves a complex interplay of genetic factors, neurobiological mechanisms, and environmental influences that contribute to the severity and progression of symptoms. Clinical manifestations are diverse, encompassing various seizure types, cognitive and behavioral impairments, and developmental delays. Current therapeutic strategies include pharmacological treatments, nonpharmacological interventions, and emerging therapies such as gene and stem cell therapy. Despite advancements, significant challenges and limitations remain, highlighting the need for ongoing research and innovation. This review synthesizes existing knowledge, identifies research gaps, and proposes future directions, emphasizing the potential for personalized medicine to improve patient outcomes and quality of life.

## Introduction and background

Epileptic encephalopathy (EE) refers to a group of disorders characterized by severe epilepsy and associated cognitive, behavioral, and neurological impairments. These disorders typically begin in early childhood and are marked by frequent, often refractory, seizures that contribute to developmental delays and intellectual disabilities. Unlike other forms of epilepsy, where seizures are the primary concern, EEs involve a dynamic interplay between ongoing epileptic activity and the progressive deterioration of cognitive and motor functions [1]. This deterioration is not solely a consequence of seizures but is also a direct result of the underlying pathological processes affecting the brain. The continuous epileptic activity exacerbates the decline in neurological function, leading to a severe and often debilitating condition for affected children [2].

Understanding the pathogenesis of EE is crucial for developing effective treatments and improving patient outcomes. The complex interplay of genetic, neurobiological, and environmental factors that contribute to these disorders necessitates a comprehensive approach to research and clinical practice. Genetic mutations, particularly those affecting ion channels and neurotransmitter receptors, play a significant role in the development of these conditions [3]. Additionally, neurobiological factors such as abnormal brain development and connectivity further complicate the clinical picture. Environmental influences, including prenatal and perinatal factors, also contribute to the onset and progression of EE. By elucidating the underlying mechanisms, researchers and clinicians can identify potential therapeutic targets and develop strategies to mitigate the impact of the disease on cognitive and developmental functions [4].

This comprehensive review aims to provide a detailed examination of the current insights into the pathogenesis and therapeutic strategies for EE. It will explore the genetic and neurobiological mechanisms that drive the disorder, discuss the various clinical manifestations, and evaluate the effectiveness of existing and emerging treatments. The review will cover the latest advancements in genetic research, highlighting how specific mutations contribute to the phenotype of EE. It will also delve into neuroimaging studies that reveal abnormal brain structures and functions associated with the disorder. Furthermore, the review will assess the efficacy of pharmacological and nonpharmacological treatments, including antiepileptic drugs (AEDs), dietary therapies, and surgical interventions. Additionally, the review will highlight the challenges and future directions in the field, emphasizing the need for ongoing research and innovation. By providing a comprehensive overview, the goal is to inform clinical practice and guide future research efforts, ultimately improving the lives of those affected by EE.

## Review

Pathogenesis of EE

The pathogenesis of EE involves a multifaceted interaction of genetic, neurobiological, and environmental factors. Genetic factors contribute significantly to 30%-50% of EE cases, with common genetic causes including mutations in ion channel genes such as SCN1A, KCNQ2, and KCNA2, which disrupt the balance between neuronal excitation and inhibition. Other implicated genes affect synaptic transmission, ligand-gated ion channels, and neuronal development. Genetic factors can lead to EE through both gain-of-function and loss-of-function mechanisms [5]. EE is characterized by an imbalance between neuronal excitation and inhibition in the brain, often resulting from the dysregulation of ion channels and synaptic proteins. Abnormal neuronal firing patterns and epileptiform activity contribute to global developmental delays and cognitive impairment. Seizures themselves can exacerbate neurological dysfunction through mechanisms like excitotoxicity and metabolic disturbances [6]. Environmental factors such as hypoxia, infection, and traumatic brain injury can also trigger EE, particularly in individuals with genetic predispositions. Epigenetic influences, such as DNA methylation and histone modifications, may interact with genetic variants to influence the onset and severity of the disease [7]. EE is frequently associated with comorbidities such as intellectual disability, autism spectrum disorder, and movement disorders. Early onset of seizures, high seizure frequency, and resistance to treatment are major risk factors for poor developmental outcomes. Further research is essential to fully understand these mechanisms and develop more targeted and effective therapies [8].

Clinical manifestations

Seizure Types and Characteristics

**Figure 1:**
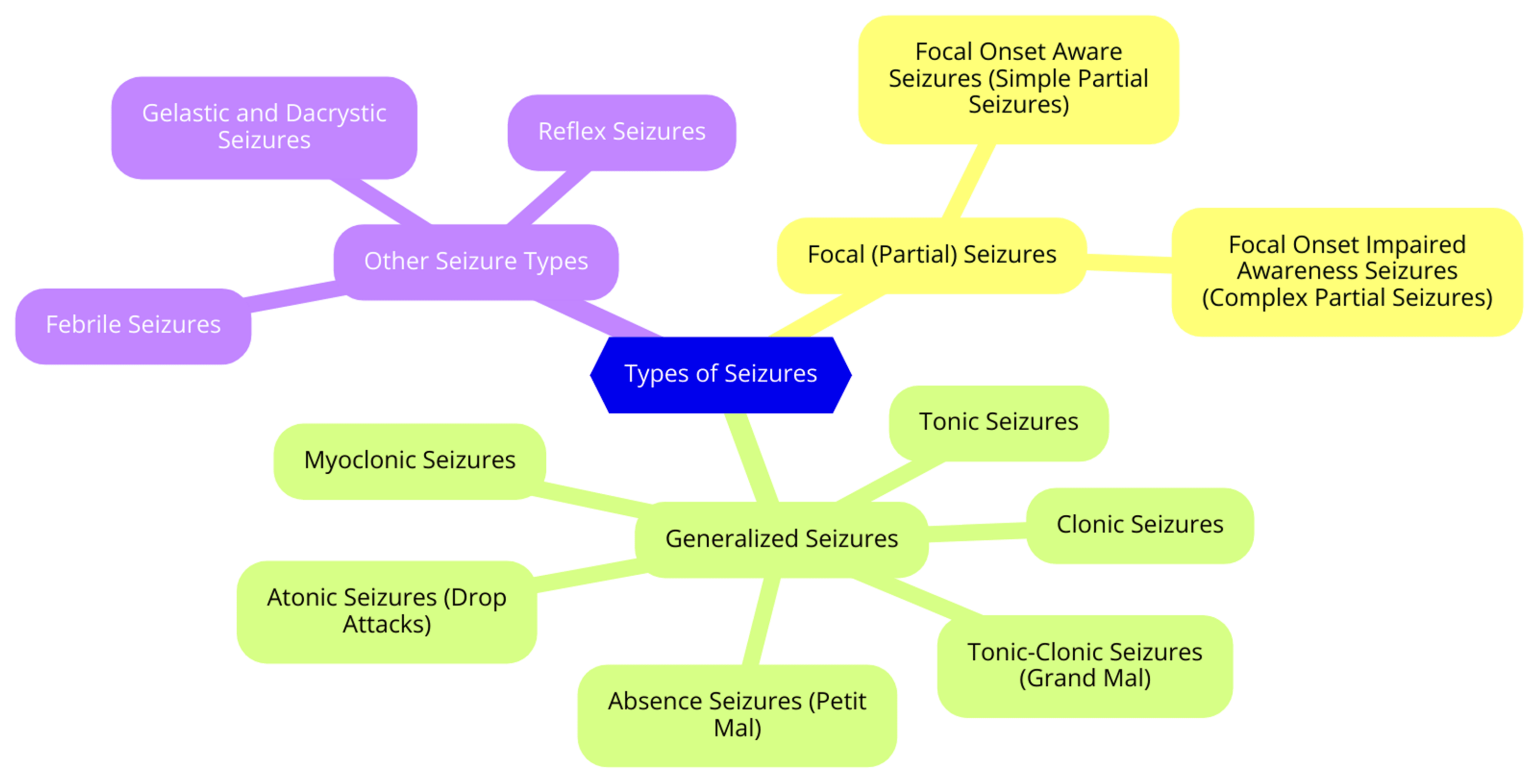
Types of seizures Image credit: Dr. Rajvardhan Patil

Seizures are categorized into several types based on their onset and characteristics. The main classifications include generalized seizures and focal seizures. Generalized seizures affect both sides of the brain and can lead to impaired consciousness or loss of consciousness. They are further subdivided into six types. Tonic-clonic (grand mal) seizures involve stiffening and jerking movements, loss of consciousness, and possible incontinence or involuntary actions. Clonic seizures feature rhythmic muscle spasms, typically affecting the face, neck, and arms. Tonic seizures cause muscle stiffening, potentially resulting in falls if the person is standing. Atonic seizures (drop attacks) are marked by sudden loss of muscle tone, resulting in falls and potential injuries. Myoclonic seizures are brief, shock-like muscle jerks that may occur in clusters. Absence (petit mal) seizures are brief episodes of staring or daydreaming, often without loss of consciousness [9]. Focal seizures originate in one part of the brain and can spread to other areas. They are further classified into two main types. Simple focal seizures do not impair consciousness and may involve symptoms such as muscle jerking, unusual sensations, or feelings of déjà vu. Complex focal seizures partially or completely impair consciousness and may present with symptoms like confusion, blank staring, or repetitive movements [10]. There is also a category of unknown-onset seizures, which cannot be definitively classified as either focal or generalized due to insufficient medical information or normal EEG results. Understanding the different types of seizures is essential for accurate diagnosis and effective treatment. Each type exhibits distinct symptoms and characteristics, enabling healthcare professionals to tailor personalized treatment plans for individuals with epilepsy [10]. Types of seizures are shown in Figure 1.

Cognitive and Behavioral Impairments

Cognitive impairment is a prevalent and significant co-occurring condition in EEs, often worsening progressively. Common deficits encompass memory loss, cognitive slowing, and attention deficits that impair daily functioning. This impairment can be permanent, stemming from the underlying epilepsy, or dynamic, arising due to seizures, interictal epileptiform activity, or the effects of AEDs [11]. Behavioral issues such as anxiety, conduct disorders, and autism are disproportionately prevalent among individuals with EEs. These psychiatric comorbidities can be as distressing as the seizures themselves, significantly reducing quality of life. Contributing factors to cognitive and behavioral impairments include structural brain lesions, uncontrolled seizures, effects of antiepileptic medications, and pre-existing mental abilities. Seizure frequency is a critical predictor, with higher frequencies correlating with more severe cognitive and behavioral deficits [12]. Thorough clinical assessment and neuropsychological evaluation are crucial for effectively diagnosing and managing these comorbidities. Addressing cognitive and behavioral challenges is essential, as they often profoundly impact quality of life more than seizures alone [13].

Developmental Delays

Developmental delays refer to a child's failure to achieve expected developmental milestones compared to peers of the same age. These delays affect learning, motor skills, communication, and social development [14]. Several factors can contribute to developmental delays. Genetic conditions like Down syndrome, metabolic disorders, and birth complications are common causes. Environmental influences such as lead exposure, inadequate nutrition, or prenatal exposure to alcohol and drugs also play a role. Other factors include medical conditions, brain injuries, and severe psychosocial trauma, although the exact cause is often unidentified [15]. Signs and symptoms of developmental delays include slower learning and development compared to peers, delays in achieving motor milestones, difficulties in communication and social interaction, below-average IQ scores, speech and logical thinking challenges, and struggles with everyday tasks. These issues significantly impact a child's ability to succeed in school and daily life [16]. Fortunately, various therapies and interventions can assist children with developmental delays. Physical therapy targets gross motor skills, while occupational therapy addresses fine motor skills, sensory processing, and self-care abilities. Speech and language therapy, early childhood special education, and behavioral therapy can also be beneficial. Early diagnosis and personalized interventions are crucial to help these children maximize their potential despite a cure for underlying conditions [17].

Diagnostic Criteria and Tools

The diagnosis of EE involves a thorough assessment using a variety of diagnostic criteria and tools. Initially, doctors gather a detailed medical history, inquiring about the patient's overall health, seizure patterns, and events surrounding seizures. A neurological examination evaluates brain and nervous system function [18]. Electroencephalography (EEG) is pivotal in diagnosing EE. This test records the brain's electrical activity through electrodes placed on the scalp, detecting abnormal brain wave patterns associated with seizures and aiding in identifying seizure type and origin. If a standard EEG yields normal results, prolonged video-EEG monitoring spanning several days may be necessary to capture seizure events [19]. Brain imaging techniques, including MRI, computed tomography (CT), positron emission tomography (PET), and single-photon emission computed tomography (SPECT) scans, offer detailed images of the brain's structure and function. These scans can reveal structural abnormalities, tumors, or changes in blood flow that may trigger seizures. Functional MRI (fMRI) can pinpoint brain regions responsible for functions like speech and memory, which seizures may affect [20]. Blood tests are often conducted to rule out other potential causes of seizures, such as infections, electrolyte imbalances, or toxins. A complete blood count (CBC) checks for signs of infection, while blood chemistry tests assess kidney function and electrolyte levels [21]. Neuropsychological testing evaluates cognitive abilities such as memory, language skills, and problem-solving capabilities. This assessment helps gauge the impact of seizures on brain function and assists in making informed treatment decisions. Diagnosing EE involves synthesizing information from the patient's medical history, seizure characteristics, EEG findings, neuroimaging results, and other diagnostic tests. Identifying the specific epilepsy syndrome and underlying causes is critical for tailoring treatment and predicting outcomes [22].

Current therapeutic strategies

Pharmacological Treatments

Current pharmacological treatments for EEs primarily focus on controlling seizures and normalizing abnormal brain activity, but their effectiveness is often limited. AEDs form the cornerstone of treatment, although many patients do not achieve seizure control even with multiple medications. Commonly prescribed AEDs include valproate, levetiracetam, lamotrigine, ethosuximide, topiramate, and zonisamide. However, certain AEDs like carbamazepine, phenytoin, and phenobarbital can exacerbate seizures and EEG abnormalities in EEs and are generally avoided [23]. Hormonal therapies such as adrenocorticotropic hormone (ACTH) and corticosteroids may be used after standard AEDs fail. Steroids, for instance, have shown cognitive and EEG improvement in 81% of cases in meta-analyses, compared to 49% with traditional AEDs. High-dose benzodiazepines like diazepam have also demonstrated efficacy, particularly in conditions like Landau-Kleffner syndrome and continuous spike-wave in slow sleep (CSWS), with a meta-analysis reporting improvement in 68% of cases [24]. Additional therapies, such as the ketogenic diet and intravenous immunoglobulin (IVIG), may provide benefits in some cases. Vigabatrin is particularly effective for treating infantile spasms associated with tuberous sclerosis complex [25]. Despite these treatment options, the long-term outlook remains uncertain, with only 10%-44% of patients achieving normal language and cognitive function. Further research is crucial to develop more effective therapies capable of preventing or reversing the severe neurological consequences of these disorders [26].

Nonpharmacological Interventions

Nonpharmacological interventions for EE encompass a range of approaches, including dietary, surgical, neurostimulation, psychological, and complementary therapies [27]. Dietary therapies, such as the ketogenic diet, have demonstrated safety and effectiveness in managing drug-resistant epilepsies in children. This high-fat, low-carbohydrate diet induces ketosis, which may help in controlling seizures. For carefully selected patients, epilepsy surgery can be considered to remove the seizure focus or disconnect pathways responsible for seizures, although this procedure carries risks of physical and psychological sequelae [28]. Neurostimulation therapies, such as vagus nerve stimulation (VNS), involve an implanted device that delivers electrical stimulation to the vagus nerve, potentially reducing seizures in some patients with drug-resistant EE. Psychological and behavioral therapies, including relaxation techniques and biofeedback, may also be beneficial by reducing stress and modifying brain activity patterns toward normalization, which could improve seizure management [29]. Complementary therapies, like acupuncture and herbal remedies, are occasionally used, but their efficacy in EE is supported by limited evidence. It is important to note that most nonpharmacological interventions should complement rather than replace standard medical treatments with anti-seizure medications (ASMs). Further research is essential to assess the safety and effectiveness of these approaches, especially in combination with pharmacological therapies, for managing this challenging condition [30].

Novel and Experimental Therapies

EE is a severe pediatric condition characterized by seizures that resist medical treatment and often lead to widespread developmental delays. The pathogenesis of EE involves an imbalance in brain excitation and inhibition, typically stemming from dysregulated voltage-gated ion channels, synaptic transmission-related proteins, and ligand-gated ion channels. Genetic factors contribute significantly, with up to 50% of cases having a genetic origin [31]. Current therapeutic strategies for EE focus primarily on managing seizures, restoring abnormal brain activity, and developing precision therapies. AEDs are the main treatment approach, but their effectiveness is limited, and many patients do not achieve seizure control even with multiple medications. Additional methods include steroids, immunoglobulins, the ketogenic diet, and, in some cases, surgery [32]. Researchers are exploring novel and experimental therapies to address the persistent challenges in treating EE. Precision therapeutics that target specific molecular mechanisms, such as modulating ion channel function, have shown promise in animal models. Gene therapy and cell-based treatments to replace dysfunctional inhibitory neurons are also under investigation. Emerging strategies include anti-inflammatory therapies and the use of cannabinoids like cannabidiol [33]. Despite advancements in understanding EE's genetic and molecular basis, developing effective, targeted therapies remains a significant hurdle. Continued research efforts and collaborative initiatives are crucial to translate these experimental approaches into viable treatment options for patients affected by these debilitating disorders [34].

Rehabilitation and Supportive Care

For patients living with EEs, rehabilitation and supportive care are integral to enhancing quality of life, maximizing function, and minimizing complications. While these conditions are incurable, the focus shifts toward managing their multifaceted challenges. Allied health therapies such as occupational therapy, physiotherapy, and speech therapy are crucial in addressing developmental delays, movement disorders, and communication impairments [35]. These therapies are tailored to each patient's needs and are essential for promoting independence and improving overall well-being. Managing individual seizure triggers and risks is a critical component of rehabilitation. This involves identifying and avoiding specific triggers such as heights, water, or fire hazards and implementing necessary safety measures to protect the patient [36]. Addressing common comorbidities like intellectual disability, autism spectrum disorder, behavioral issues, sleep disturbances, and respiratory problems is also vital. This comprehensive approach often requires collaboration among neurologists, pediatricians, nurses, therapists, social workers, and caregivers to provide holistic care. Providing assistive devices for mobility, communication, and daily activities is another significant aspect of rehabilitation. Devices such as wheelchairs, walkers, or communication aids like picture symbols are used to support independence and communication skills. Optimizing nutrition and managing gastrointestinal issues such as feeding difficulties are essential for care. Preventing and addressing complications like orthopedic problems and sudden unexpected death in epilepsy (SUDEP) are also paramount considerations [37]. A coordinated, multidisciplinary approach ensures that patients receive comprehensive care tailored to their needs, aiming to enhance their quality of life and functional outcomes.

Challenges and future directions

The limitations of current therapies for treating EEs are profound and multifaceted. ASMs often demonstrate limited efficacy, especially since these conditions are frequently resistant to medical treatment from the onset. Even with combinations of ASMs, many patients fail to achieve adequate seizure control. Unfortunately, neurological impairments such as intellectual disability, movement disorders, and respiratory issues often persist despite treatment, with any deficits caused by seizures typically being permanent, even if the seizures eventually cease [38]. Another significant constraint is the lack of definitive evidence to guide treatment decisions. There is a scarcity of high-quality clinical trials establishing evidence-based treatment protocols, forcing clinicians to rely on limited data when managing these devastating conditions. Current targeted therapies, such as gene therapy approaches, face challenges such as administration route, timing of intervention, specificity to cell types, packaging constraints, and insufficient long-term safety data. Moreover, translating the functional effects of genetic variations observed in vitro to human clinical applications remains a considerable challenge [39]. Furthermore, the intricate neurobiological mechanisms linking specific mutations to seizure development are still not fully understood despite genetic advancements. Extensive research is essential to comprehend the complex pathophysiology underlying EEs better. While there has been some progress, significant limitations persist in effectively treating these conditions. Ongoing research endeavors are critical to developing more precise and effective therapies aiming to improve long-term outcomes for children affected by EEs [40].

Implications for clinical practice and policy

The implications for clinical practice and policy in treating EEs are multifaceted and underscore several critical considerations. Early diagnosis and immediate intervention are paramount, as timely treatment may enhance cognitive outcomes in these severe conditions. It is imperative for experienced specialists to swiftly devise treatment strategies upon identifying EEs and recognize the urgency of intervention [35]. However, clinicians are currently constrained by the limited efficacy of available therapies. Despite efforts with ASMs, hormone therapy, and immune modulators, many cases exhibit inadequate response to treatment. The absence of standardized treatment guidelines based on robust clinical evidence necessitates clinicians to rely on limited data, emphasizing the urgent need for more comprehensive clinical research [41]. Continued research aimed at a deeper understanding of the underlying pathogenic mechanisms, particularly focusing on genetic factors and ion channel dysregulation, remains a priority. This knowledge is crucial for developing targeted therapies tailored to specific EE syndromes, such as the use of retigabine for KCNQ2-related epilepsy. These personalized approaches hold promise for optimizing treatment outcomes [42]. Moreover, conducting larger-scale, high-quality clinical trials is essential to establish evidence-based guidelines for managing these debilitating conditions. While anecdotal evidence and small series suggest that reducing EEG ictal and/or interictal activity might improve cognitive and behavioral outcomes, the efficacy of such approaches remains contentious due to the absence of conclusive data from well-designed studies [43]. Advancing clinical practice and policy in treating EEs necessitates early intervention, personalized treatment strategies informed by robust research, and the establishment of evidence-based guidelines through rigorous clinical trials. These efforts are critical to enhancing the quality of care and outcomes for individuals affected by these challenging neurological disorders

## Conclusions

In conclusion, the comprehensive understanding of EE's pathogenesis and therapeutic strategies underscores the complexity and multifaceted nature of this debilitating group of disorders. The interplay of genetic mutations, neurobiological mechanisms, and environmental influences contributes to the severity and progression of symptoms, emphasizing the need for a tailored approach to diagnosis and treatment. Current therapeutic options, ranging from pharmacological treatments to innovative interventions like gene and stem cell therapy, offer hope but highlight the limitations and challenges in achieving optimal patient outcomes. As research advances, the potential for personalized medicine and novel therapies grows, promising more effective and individualized treatment plans. This review synthesizes current knowledge and identifies critical gaps and future directions, advocating for a collaborative effort in research and clinical practice to enhance the quality of life for individuals affected by EE.
